# Role of WWOX and NF-κB in lung cancer progression

**DOI:** 10.1186/2213-0802-1-15

**Published:** 2013-11-14

**Authors:** Szu-Jung Chen, Shenq-Shyang Huang, Nan-Shan Chang

**Affiliations:** 1 Institute of Molecular Medicine, National Cheng Kung University College of Medicine, Tainan, Taiwan; 2 Department of Neurochemistry, New York State Institute for Basic Research in Developmental Disabilities, Staten Island, NY USA; 3 Department of Neuroscience and Physiology, SUNY Upstate Medical University, Syracuse, NY USA

**Keywords:** WWOX, Tumor suppressor, Tumor necrosis factor, NF-κB, Lung cancer

## Abstract

It is generally agreed that the pro-inflammatory, pro-survival transcription factor NF-κB is a tumor promoter. Tumor necrosis factor alpha (TNF-α or TNF) mediates NF-κB activation. Tumor suppressor WWOX (FOR or WOX1) is a downstream effector of the TNF signaling. Thus, activation of both WWOX (FOR or WOX1) and NF-κB may occur during TNF signaling and/or under stress conditions. Indeed, the first WW domain of WWOX induces the activation of NF-κB-responsive promoter without TNF participation. It appears that WWOX counteracts with NF-κB in regulating cell survival and death. For example, WWOX becomes activated with Tyr33 phosphorylation and relocates together with NF-κB and many transcription factors to the nucleus to cause neuronal death in sciatic nerve-transected rats. While WWOX is frequently lost in lung cancer and many other cancers, NF-κB activation-induced cancer promotion probably requires WWOX-independent signaling networks to induce expression of pro-survival factors. The antagonistic role of WWOX and NF-κB in the regulation of lung cancer progression is discussed.

**Electronic supplementary material:**

The online version of this article (doi:10.1186/2213-0802-1-15) contains supplementary material, which is available to authorized users.

## Inflammatory TNF-α/NF-κB pathway in lung cancer

Inflammatory cytokines are involved in the pathogenesis of lung cancer progression [1]. The cytokines may drive the activation of nuclear transcription factors, which fosters the generation of favorable microenvironment for sustaining the growth of cancerous cells. As a pro-inflammatory cytokine, tumor necrosis factor alpha (TNF-α or TNF) participates in many events that lead to oxidative stress, vasodilatation, edema formation, and fever. Prosurvival transcription factor nuclear factor-κB (NF-κB), for example, is a downstream effector of the TNF-α pathway, and is being regarded as a crucial factor during cancer initiation and progression [2, 3]. NF-κB could orchestrate the signals from different pathways, and interacts with signaling proteins such as transcription factors STAT3 and p53 or the ETS related gene ERG [4]. The transcriptional activity of NF-κB could also be affected by kinases from other signal pathways, such as GSK3-β, p38, and PI3K [4]. Networking analysis revealed that NF-κB directly or indirectly interacts with BCL-3 (B-cell CLL/lymphoma 3), ESR-1 (estrogen receptor 1), NR3C1 (nuclear receptor subfamily 3, group C, member 1; glucocorticoid receptor), ELF1 (E74-like factor 1; ets domain transcription factor) and many more. Indeed, there are many binding partners with NF-κB [4, 5], the molecular mechanisms underlying NF-κB integration with these signals is largely unknown.

NF-κB is a homo- or heterodimeric complex from a composition of the Rel-like domain-containing proteins RELA/p65, RELB, NFKB1/p105, NFKB1/p50, REL and NFKB2/p52 [4, 5]. The heterodimeric p65-p50 complex can be found in cells in most cases. NF-κB is frequently localized or sequestered in the cytoplasm. This is mainly due to the regulatory effect of inhibitor protein IκBα. IκBα binds and masks the nuclear localization signals of NF-κB. IκBα can be phosphorylated at Ser32 and Ser36 by a specific IκB kinase (IKK) complex and is then degraded in a ubiquitin/proteasome-dependent manner [4–6]. Degradation of IκBα results in activation or nuclear accumulation of NF-κB. The IKK complex is composed of two catalytic components IKKα and IKKβ and a regulatory subunit IKKγ (NEMO). The aforementioned event has been considered as a canonical pathway of NF-κB activation. For the noncanonical NF-κB activation, IKKα binds and phosphorylates a p100 complex, in which p100 undergoes ubiquitination and is degraded to p52. NF-κB interacts with p52 for relocating to the nucleus and regulates gene transcription [4–6]. Tax, a viral regulatory protein from human T-cell lymphotropic virus type 1 (HTLV-1), is a key inducer of the NF-κB activation pathway and may contribute to the pathogenesis of viral oncogenesis [7, 8].

Cancer initiation and progression can be considered as a chronic inflammatory process. Constitutive activation of NF-κB is frequently shown in cancer cells, and the activated NF-κB affects cancer cell growth, progression and metastasis. In lung cancer and chronic obstructive pulmonary disease (COPD), the NF-κB pathway is linked to the inflammatory signaling, oxidative stress response, and glycolysis and gluconeogenesis pathways, as revealed by proteomic analyses [9]. Bromodomain-containing protein 4 (Brd4) maintains the constitutively active NF-κB in lung cancer cells by binding acetylated RelA [10]. However, NF-κB activity is needed to activate immune surveillance for the anti-lung cancer response [11]. In contrast, SOD2 (Superoxide Dismutase 2, Mitochondrial) induces the activation of NF-κB and increases IKKβ transcription in lung adenocarcinoma. The event favors the progression of lung cancer and confers poor prognosis in patients [12].

### Unconventional inhibitors of NF-κB

The BCL-3 subfamily protein physically interacts with NF-κB in the nucleus and thereby functionally controls its transcriptional activity [13, 14]. Like BCL-3, atypical inhibitors of NF-κB have been identified such as IκBζ long, IκBζD, IκBζ short, IκB_NS_, and IκBη [13]. These proteins possess common structural domains such as ankyrin repeats, transactivation domains, and nuclear localization signal [13]. Binding of these proteins with DNA-anchored NF-κB modulates its transcriptional function. These atypical inhibitors are not subjected to degradation even after NF-κB activation by stimuli such as lipopolysaccharide or Interleukin 1 beta (IL-1β). Instead, their levels are raised intracellularly.

Additionally, we have determined that Zfra (**z** inc **f** inger-like protein that **r** egulates **a** poptosis) regulates TNF-α-mediated cell death by interacting with receptor adaptor protein TRADD (TNF receptor-associated death domain protein) and downstream JNK (c-Jun *N*-terminal kinase), NF-κB, and WWOX or WOX1 (WW domain-containing oxidoreductase) [15, 16]. Transiently overexpressed Zfra sequesters NF-κB (p65), WWOX, p53 and phospho-ERK (extracellular signal-activated kinase) in the cytoplasm, and that TNF-α or UV light could not effectively induce nuclear translocation of these proteins. This study directly demonstrated a cytoplasmic control of NF-κB activation by Zfra. Interestingly, missense mutations of caspase-8 activate NF-κB signaling in cancer cells [17].

### TNF-α signaling and programmed cell death

TNF-α-mediated cell death is one of the biological events in development, aging, and metabolic turnover. In general, cells are highly organized in multicellular organism. The number of cells in each organism is strictly controlled during development. If cells are damaged or aged, they commit suicide by promoting an intracellular death program - the so-called programmed cell death. Apoptosis is one of the physiological death in a “programmed” manner. It is generally agreed that apoptotic cells undergo membrane blebbing and then shrinkage, nuclear condensation, nuclear membrane disassembly, and chromosomal DNA fragmentation [18–21]. Unfortunately, studies by time-lapse microscopy fail to support all types of apoptosis acting in this typical manner. Apoptotic bodies, which are generated from the whole cell membrane blebbing and fragmentation, are readily phagocytosed or cleared up by macrophages. Intracellular machinery responsible for the programmed cell death goes through a family of cysteine proteases, named caspases (also known as cysteine-aspartic proteases or cysteine-dependent aspartate-directed proteases) [20, 21]. Caspases also participate in necrosis and inflammation. Caspases possess a cysteine at the active site, and once activated, target proteins are cleaved at specific aspartic acids [20, 21]. That is to execute the programmed cell death.

Numerous intrinsic and extrinsic stimuli lead to signaling cascades that culminate in programmed cell death [22]. In the extrinsic or death receptor pathway, signals from extracellular environment such as TNF-α, toxins, hormones, growth factors, and/or cytokines, interact with membrane receptors so as to instigate the downstream cascade of protein/protein interactions and thereby generate biological effects from gene transcription and new protein production. These extrinsic signals may positively or negatively affect the execution of programmed cell death or apoptosis [23, 24]. Several extrinsic signals, including Fas ligand [25], Apo2 ligand, and TNF-related apoptosis-inducing ligand (Apo2L/TRAIL), instigate apoptosis by binding to cognate receptors such as Fas, death receptor 4 (DR4), and death receptor 5 (DR5) [26, 27]. By the same token, TNFα-induced cell death is mediated through specific cell surface receptors TNF-R1 and TNF-R2 [21, 23].

In the intrinsic or mitochondrial pathway, damage to chromosomal DNA by oxidative stress, UV irradiation, or therapeutic chemicals may initiate the intracellular death pathway [28–30]. As a consequence, caspase activation occurs, which leads to cytochrome c release from the mitochondria into the cytosol. Cytochrome c binds and causes the aggregation of the adaptor protein Apaf-1 (Apoptotic protease activating factor 1), activation of procaspase-9 and subsequent nuclear DNA damage [28]. Cumulative evidence has shown that inhibitors of histone deacetylase activate the intrinsic/mitochondrial pathway for leading to cell death via upregulation of a number of proapoptotic BH3-only Bcl-2 family genes, including Bim, Bid, and Bmf [29].

### Pro-apoptosis versus anti-apoptosis in TNF-α signaling

TNF-α initiates two counteractive signal pathways - one for pro-apoptosis and the other for anti-apoptosis [2]. How TNF-α acts to determine the desired outcome, either cell death or survival, is largely unknown. Two different types of transmembrane TNF receptors have been identified. Upon stimulation of TNF-R1 with TNF-α, death domain-containing protein TRADD (TNF receptor-associated death domain) becomes associated with the receptor and then recruits another death domain protein via the death domain/death domain binding. These proteins include FADD (Fas-associated death domain protein), RIP1 (receptor-interacting protein 1), and caspase 8 [2]. When the adaptor proteins are dissociated from TNF-R1, caspase-8 becomes activated for leading to the downstream apoptosis event including nuclease-induced chromosomal DNA fragmentation and nuclear condensation.

The kinase RIP1 appears to be a deciding factor for cell survival or death during TNF-R1 signaling [30, 31]. To turn off TNF-R1 signaling-induced cell death, ubiquitin-editing enzyme A20 binds Itch via a regulatory TAX1BP1 to prevent recruitment and inactivation of RIP1 [30]. Additionally, post TNF-α stimulation, RIP1 is conjugated with ubiquitin chains via K63 and functions as a scaffold to build signaling complexes and activate kinases for protective gene expression [31]. Also, K48-dependent linear ubiquitination of RIP1 by LUBAC ubiquitin ligase complex leads to degradation by the proteasome-dependent mechanism, thus enhancing the activation of NF-κB pathway and cell survival [32].

### TRAF2: an inhibitor of TNF-α-induced cell death

TNF-induced apoptosis can be turned off by activating NF-κB through TNF-α receptor-associated factor 2 (TRAF2) [33]. The molecular action is that TRADD directly interacts with TRAF2, and then activates NF-κB for inducing the anti-apoptotic event [34–38]. In UV-activated cell apoptosis, TRAF2 promotes cell survival via activating NF-κB and inhibiting p53 from binding to the mitochondria and blocking the release of cytochrome C [39, 40]. Additionally, TRAF2 controls the expression of lung Krüppel-like factor (LKLF) via the mitogen-activated protein kinase p38 pathway to counteract TNF-induced apoptosis [41]. The role of TRAF2 in the lung cancer development and progression is largely unknown. It has been determined that endogenous phosphorylated TRAF2 and ribosomal protein S3 confer resistance to irradiation-mediated death of non-small cell lung cancer (NSCLC) cells [42]. TRAF2 and RIP1 also participate in suppression of TRAIL-induced autophagy via activation of JNK1 [43].

**Figure 1:**
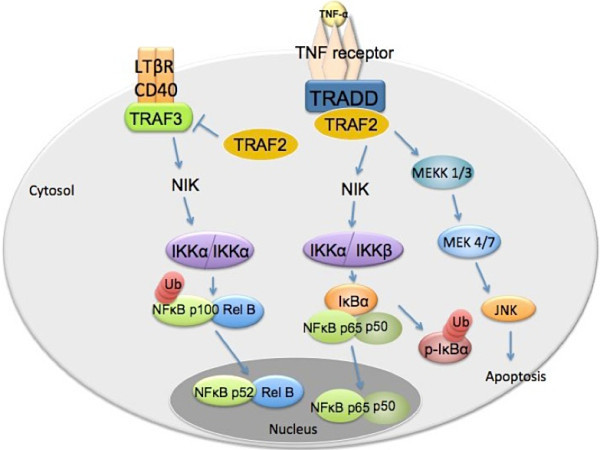
**TRAF2 signal pathway networks.** TRAF2 promotes canonical NF-κB pathway, and suppresses noncanonical NF-κB pathway [45, 46, 50, 51]. TNF-R1 activates the canonical NF-κB pathway via recruiting TRADD, which in turn binds adaptor proteins TRAF2, TRAF5 and RIP1 [44]. These adaptor protein complexes activate the IKK (IκB kinase) proteins for NF-κB (p65/p50 heterodimer) activation, following phosphorylation and degradation of the inhibitory protein IκBα [45, 46]. On contrary, LTβR (Lymphotoxin β receptor) promotes noncanonical NF-κB signaling pathway bypassing activation of the pro-apoptotic caspase cascades, but directly recruiting TRAF2 and TRAF3. IKKα homodimers are then activated through the upstream NIK (NFκB inducing kinase). IKKα induces NF-κB p100 precursor phosphorylation, followed by partially processing to a p52 form for leading to noncanonical NF-κB (p52/ RelB heterodimer) activation [45, 46].

In the canonical TNF/NF-κB pathway, activated TNF-R1 recruits TRADD, which in turn binds adaptor proteins FADD, TRAF2, TRAF5 and RIP1 [44] (Figure 1). These adaptor protein complexes activate the IKK kinases for IκBα degradation and NF-κB activation [45, 46]. Although activated TRADD triggers apoptotic signaling by recruiting FADD and caspase-8 and prolonging JNK activation, NF-κB activation leads to the expression of anti-apoptotic proteins such as cFLIP (cellular FLICE (caspase-8)-like inhibitory protein) and cIAP (cellular inhibitors of apoptosis), which block caspase-8 activation [47, 48]. Lymphotoxin β receptor (LTβR) promotes noncanonical NF-κB signaling pathway bypassing activation of pro-apoptotic caspase cascades, but directly recruits TRAF2 and TRAF3, which then activates IKKα homodimers through NIK (NF-κB inducing kinase) (Figure 1). IKKα induces NF-κB p100 precursor phosphorylation, and the protein is processed to a p52 form for causing noncanonical NF-κB (RelB/p52 heterodimer) activation [45, 46].

TRAF2 is a member of the TRAF family, and is responsible for activating canonical NF-κB pathways. In mouse embryonic fibroblasts (MEF), TRAF2 and TRAF5 double knockout suppresses TNFα-induced NF-κB activation. Conditional knockout of TRAF2 in B cells causes noncanonical NF-κB pathway activation [49]. That is, TRAF2 promotes canonical NF-κB pathway, but suppresses noncanonical NF-κB pathway [45, 46, 50, 51].

**Figure 2:**
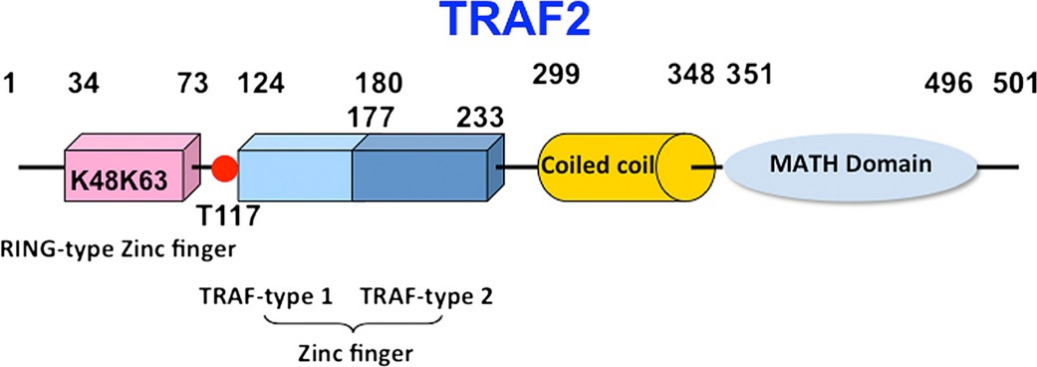
**A schematic structure of TRAF2.** The full-length TRAF2 is composed of 501 amino acids (molecular weight 55 kDa). The RING-type zinc finger domain possesses an E3 ubiquitin-protein ligase activity through Lys63 (K63) or Lys48 (K48) activation. The coiled coil domain mediates TRAF2 homo- or hetero-oligomerization, and phosphorylation at Thr117 (T117) is needed for NF-κB activation. The MATH/TRAF domain binds to receptor cytoplasmic domains.

TRAF2 is a 501-amino-acid protein, possessing a RING-type zinc finger domain, a coiled coil domain, and a MATH/TRAF domain (Figure 2). The RING-type zinc finger domain is associated with an E3 ubiquitin-protein ligase activity. The coiled coil domain mediates TRAF2 homo- or hetero-oligomerization, and a phosphorylation site at Thr117 is important for NF-κB activation. The MATH/TRAF domain binds to receptor cytoplasmic domains. The RING-type zinc finger domain activates both JNK/c-Jun and IKK/NF-κB pathways [52]. Deletion of RING-type zinc finger domain of TRAF2 (TRAF2-ΔR) has been widely used as a dominant negative inhibitor of TNF-α-induced activation of JNK and IKK. Stable expression of TRAF2-ΔR in TRAF2 and TRAF5 double knockout cells efficiently inhibits TNF-α-induced prolonged activation of JNK, but fails to suppress cell death. Moreover, stable expression of TRAF2-ΔR in TRAF2 and TRAF5 double knockout cells does not suppress the noncanonical NF-κB pathway [53].

### Tumor suppressor WWOX (FOR or WOX1) in signaling

**Figure 3:**
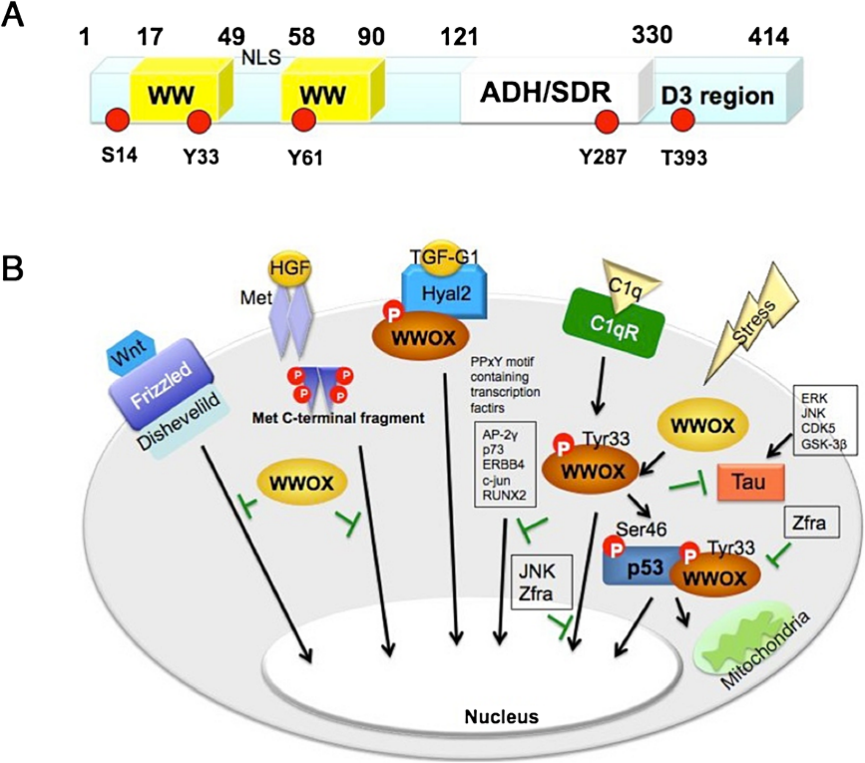
**WWOX/WOX1 and signaling networks. (A)** A schematic structure of WWOX/WOX1 (414 amino acids; molecular size 46 kDa) is shown. Two *N*-terminal WW domains are encoded by exon 1–4 of the *WWOX* gene, and the ADH/SDR domain by exon 4–8. There is a NLS (nuclear localization signal) between the two WW domains, and a NSYK motif in the ADH/SDR domain. The *C*-terminal tail D3 domain possesses an apoptotic function. The conserved phosphorylation sites are indicated [54–63]. **(B)** WWOX interacts with binding proteins via PPXY motifs. Protein of these categories are p73, activator protein 2γ (AP-2γ), ErbB4, ezrin, small integral membrane protein of the lysosome/late endosome (SIMPLE), c-Jun [59–63]. WWOX is involved in the Wnt/β-catenin pathway and the HGF (hepatocyte growth factor)/MET pathway. WWOX acts synergistically with p53 to mediate apoptosis, and that JNK and Zfra block the effect. Complement C1q induces an unconventional apoptosis of prostate cancer cells [59, 60, 62]. WWOX prevents neurodegeneration by inhibiting Tau hyperphosphorylation caused by ERK and GSK-3β [59, 60, 62]. Hyaluronidase Hyal-2 acts as a receptor for TGF-β1. During signaling, WWOX is activated and binds Smad4 and the protein complex is accumulated in the nucleus, which may lead to cell survival or death [59, 60, 62].

Many outstanding review articles have described the *in vitro* and *in vivo* roles of tumor suppressor WW domain-containing oxidoreductase, designated WWOX, FOR, or WOX1, in tumor suppression, metabolic disorders, immune defects, bone tumors, neurodegenerative diseases and others [54–63]. Human *WWOX* gene, containing 1 million bases with 9 exons, is located in chromosome 16q23.3–24.1. This region is known as a chromosomal common fragile site FRA16D. The encoded protein contains two *N-* terminal WW domains, a *C-* terminal short chain alcohol dehydrogenase/reductase (ADH/SDR) domain, and a D3 tail at the *C*-terminus. Additionally, an NSYK (Asn-Ser-Tyr-Lys) motif for binding with sex steroid hormones, a nuclear localization signal (NLS) (GKRKRV), and a mitochondria-targeting sequence in the ADH/SDR domain have been defined in WWOX [60–63] (Figure 3A).

WWOX is located ubiquitously within the cell. It can be found in the cytoplasm, cell membrane/cytoskeleton, organelles and nucleus [60–63]. The first WW domain binds target proteins containing the proline-rich PPXY-motif(s) during signal transduction. For example, WWOX interacts with p73, activator protein 2γ (AP-2γ), ErbB4, ezrin, small integral membrane protein of the lysosome/late endosome (SIMPLE), and c-JUN [59–63] (Figure 3B). Transiently overexpressed WWOX blocks the nuclear accumulation of p73, AP-2γ, and c-JUN *in vitro* [54, 60]. However, the observations are not true *in vivo* [54, 60, 64]. In the Wnt/β-catenin pathway, transiently overexpressed WWOX prevents nuclear import of Dishevelled [65]. Similarly, in the HGF/MET pathway, ectopic WWOX inhibits the MET *C*-terminal fragment for nuclear translocation and suppression of the downstream gene expression [66].

Moreover, WWOX is involved in stress and apoptotic responses (Figure 3B). WWOX is shown to stabilize tumor suppressor p53 via a direct binding interaction [64–70]. UV-induced WWOX activation with Tyr33 phosphorylation binds Ser46-phosphorylated p53. The binding interaction is independent of the WW domain-PPXY rule [60–63]. The p53/WWOX complex may translocate to the mitochondria and further to the nucleus to mediate apoptosis *in vitro* and *in vivo*. And, JNK (c-Jun *N*-terminal kinase) and Zfra (zinc finger-like protein that regulates apoptosis) inhibit the apoptotic response [15, 16, 68, 71–73]. Sex steroid hormones estrogen and androgen are shown to induce the complex formation of p53 and WWOX and their accumulation in the nucleus [74, 75]. Complement C1q invokes WWOX activation via an unconventional pathway of apoptosis for causing cancer cell death [57, 76]. WWOX prevents neurodegeneration by inhibiting Tau hyperphosphorylation caused by ERK and GSK-3β [54, 60, 62, 77, 78]. Hyaluronidase Hyal-2 acts as a receptor for TGF-β1. During signaling, the SDR domain of WWOX physically interacts with Hyal-2, and the resulting WWOX/Hyal-2 complex binds Smad4 and is then accumulated in the nucleus, which may increase cell survival or death both *in vitro* and *in vivo* [60, 62, 79]. A recent study has demonstrated that WWOX interacts with an oncoprotein latent membrane protein 2A (LMP2A), via the PPXY motif, in Epstein-Barr virus, and that this interaction induces activation of extracellular signal-regulated kinase (ERK), upregulation of matrix metalloproteinase 9 (MMP9), and promotion of cell invasion [80]. Overall, interaction of WWOX with p53, JNK1, Zfra, c-Jun, CREB, and many others is WWOX activation-dependent [54, 60, 62, 75]. That is, WWOX undergoes phosphorylation at Tyr33. CREB is shown to enhance the apoptotic function of WWOX [64].

Many proteins have one or 2–5 repeats of WW domains. It has been proposed that these domains can work in a coherent manner [81]. One report has demonstrated that the first WW domain of WWOX binds WW-binding protein 1 (WBP1) and WW-binding protein 2 (WBP2) signaling adaptors via PPXY motifs [82]. It is postulated that the first WW domain is responsible for the binding interaction, and the secondary WW domain fails to interact with the PPXY motifs. By site-directed mutagenesis using synthetic peptides, co-immunoprecipitation and physical approaches, the secondary WW domain appears to act as a chaperone to stabilize the first WW domain [82]. Nonetheless, the regions flanking the WW domains (e.g. SDR domain and D3 tail) were not functionally examined and the physiological relevance of WWOX-WBPs interactions is not known.

Indeed, we have determined that the SDR domain of WWOX binds tau so as to block enzyme-mediated hyperphosphorylation [77, 78]. Also, the SDR domain interacts with membrane Hyal-2 during TGF-β signaling [79]. MEK1, a mitogen-activated protein kinase, physically interacts with the SDR domain of WWOX, and that dissociation of this complex by phorbol ester induces apoptosis in leukemia cells [83].

### WWOX in tumor suppression and metabolic disorders

WWOX protein expression is frequently downregulated in invasive cancer cell types [59–63]. Suppression of WWOX in invasive cells may be due to gene mutation, deletion, translocation, hypermethylation of CpG island in the promoter region [84], and mRNA translational blockade [70]. Restoration of WWOX in lung and other cancer cells inhibits their growth and tumorigenicity *in vitro* and *in vivo* [59–62]. Although downregulation of tumor suppresser *WWOX* expression has frequently been reported in various types of cancers, *Wwox* deletion did not necessarily increase the proliferation or development of premalignant lesions, suggesting that *WWOX* is not a classical tumor suppressor gene. Interestingly, in a spontaneous mutant *lde/lde* (lethal dwarfism with epilepsy) rat strain, frame deletion of *Wwox* gene has been shown, and the rat possesses aberrant Wwox protein expression in the central nervous system and development of seizure [85]. In knockout mouse model, the animals can only survive for one month and have defects in bone metabolism, splenic atrophy, and other deficiencies [59–61, 86, 87].

Two single nucleotide polymorphisms in *MAF* (musculoaponeurotic fibrosarcoma oncogene homologue) and *WWOX* genes are associated with reduced insulin secretion and hypertension [88]. MafA and c-maf are critical for the β- and α-cells islet development as well as insulin and glucagon biosynthesis [89]. However, *WWOX* gene inhibits pancreatic islet development by inactivation the Wnt/β-catenin pathway [65].

### WWOX in lung cancer

Alterations in *WWOX* genes are associated with lung cancer development. WWOX is mapped to the chromosomal common fragile site FRA16D and several copy number variations (CNVs) are associated with this gene. It appears that loss of CNV-67048 genotypes in *WWOX* in Chinese predisposes their carriers to lung cancer [90]. Whether this affects *WWOX* gene expression and loss of exons is unknown. Similar studies also showed that the polymorphisms and haplotypes of *WWOX* gene are associated with the risk of lung cancer in southern and eastern Chinese populations [91]. Ectopic expression of wild type *WWOX* suppresses the growth human non-small cell lung cancers (NSCLCs) both in cell culture and in patients in a SDR domain-dependent manner [92, 93]. *WWOX* gene expression may be considered as a prognostic biomarker in surgically resected, early-stage NSCLC. An epigenetic regulator/polycomb group protein Bmi1 is more highly expressed in small-cell lung cancer (SCLC) than in NSCLC, and acts by blocking the expression of *WWOX* at the transcriptional level [94]. MicroRNA miR-134 targeting *WWOX* expression is associated with head and neck carcinogenesis [95]. Again, alterations in the *WWOX* gene, including hypermethylation of *WWOX* gene promoter region and mutations, may contribute to lung carcinogenesis [96]. A recent study showed that ectopic *WWOX* is able to suppress autophagy for inducing apoptosis in methotrexate-treated human squamous cell carcinoma, and that induction of *WWOX* expression in SCC is associated with cure of this cancer in patients [97].

### WWOX and NF-κB in the regulation of lung cancer growth

Substantial evidence has shown that NF-κB is both a mediator of inflammation and a promoter of carcinogenesis [98–100]. How NF-κB orchestrates inflammation toward carcinogenesis is largely unknown. A recent report showed that in the *Gprc5a* gene knockout mice, NF-κB activation occurs in airway epithelium, which ultimately leads to lung inflammation and tumorigenesis [101]. It appears that increased inflammatory autocrine and paracrine interactions contribute to carcinogenesis. Alternatively, noncanonical TBK1 and IKKɛ contribute to NF-κB activation and the inflammatory responses for carcinogenesis [100].

WWOX has been implicated in the regulation of the canonical and noncanonical NF-κB pathways in HTLV-I Tax-mediated tumorigenesis. The viral oncoprotein Tax suppresses WWOX expression by inhibition of the noncanonical NF-κB pathway [6, 8, 39, 58]. Conversely, WWOX effectively blocks Tax-induced activation of the canonical NF-κB pathway, whereas the noncanonical pathway is not affected [6, 8, 39, 58]. The observations suggest the role of the noncanonical NF-κB pathway in contributing to carcinogenesis.

It appears that WWOX can override the pro-survival effect of NF-κB to apoptosis. We have determined that overexpression of the first WW domain of WWOX induces the activation of NF-κB-responsive promoter without the participation of TNF-α *in vitro* [64]. In sciatic nerve-transected rats, Wwox becomes activated with Tyr33 phosphorylation and relocates together with NF-κB and many transcription factors to the nuclei to cause neuronal death [64]. Wwox binds activated CREB and c-Jun, but not NF-κB, as determined from both *in vivo* and *in vitro* experiments [64]. Complement C1q, which belongs to the TNF-α-like family of proteins, activates WWOX to induce cancer cell death [76]. Whether NF-κB is also activated is unknown. However, activation of NF-κB is shown during C1q stimulation of monocytes [102]. Thus, the functional relationship between WWOX and NF-κB has yet to be determined. While WWOX is frequently lost in lung cancer and many other cancers, NF-κB activation-induced cancer promotion probably requires WWOX-independent signaling networks to induce expression of pro-survival factors.

Finally, there are intriguing interactions among viral LMP2A, WWOX and NF-κB. WWOX binds LMP2A to induce ERK activation, MMP9 upregulation, and promotion of cell invasion [80]. LMP2A, together with NF-κB, protects B-cells from apoptosis by blocking B-cell receptor (BCR) signaling [103]. However, the effect of WWOX on B cell survival is unknown.

## Review and conclusions

Activation of NF-κB is crucial for inflammatory response, as well as for carcinogenesis. NF-κB is activated by both canonical and noncanonical approaches. TNF-α is mainly responsible for the canonical NF-κB activation, and LTβR and CD40 for the noncanonical NF-κB activation. Complement C1q activates both WWOX and NF-κB [57, 76, 102]. WWOX fails to directly bind NF-κB. HTLV-I Tax-mediated tumorigenesis is associated with activation of NF-κB via both canonical and noncanonical pathways, and that WWOX may interfere with this pathway [58]. Apparently, there is a functional antagonism between WWOX and NF-κB. WWOX interacts with Epstein-Barr virus LMP2A, and the interaction activates ERK. Whether NF-κB becomes activated has yet to be determined. Nonetheless, the MEK/ERK signaling is prosurvival. Binding of WWOX with LMP2A may render functional inactivation of WWOX and thereby enhances cancer growth and invasion. Conformational and functional alterations of WWOX due to binding with LMP2A are likely. Phorbol ester is known to activate ERK, and that this event may lead to NF-κB activation [104]. WWOX binds MEK and affects ERK activation, which shuts down NF-κB activation [83]. Taken together, functional antagonism between WWOX and NF-kB is likely to occur during lung cancer initiation and progression. A balance of both proteins is critical in controlling cancer formation.

## Authors’ original submitted files for images

Below are the links to the authors’ original submitted files for images. Authors’ original file for figure 1
Authors’ original file for figure 2
Authors’ original file for figure 3

## Abbreviations

WWOX: WW domain-containing oxidoreductase
NF-κB: Nuclear factor kappa-light-chain-enhancer of activated B cells
JNK1: cJun *N*-terminal kinase
Zfra: Zinc finger-like protein that regulates apoptosis
NSCLC: Non-small-cell lung carcinomas
SCLC: Small-cell lung cancer
ECM: Extracellular matrix
TNF-α: Tumor necrosis factor alpha
IκBα: Inhibitor of NF-κB alpha
IκBβ: Inhibitor of NF-κB beta
IKK: Kinase of inhibitor of IκB (IκBα or IκBβ).

## References

[1] Lu H, Ouyang W, Huang C (2006). Inflammation, a key event in cancer development. Mol Cancer Res.

[2] Li J, Yin Q, Wu H (2013). Structural basis of signal transduction in the TNF receptor superfamily. Adv Immunol.

[3] Zelová H, Hošek J (2013). TNF-α signalling and inflammation: interactions between old acquaintances. Inflamm Res.

[4] Hoesel B, Schmid JA (2013). The complexity of NF-κB signaling in inflammation and cancer. Mol Cancer.

[5] Niederberger E, Geisslinger G (2013). Proteomics and NF-κB: an update. Expert Rev Proteomics.

[6] Xiao G, Rabson A, Youn W, Qing G, Qu Z (2006). Alternative pathways of NF-kappaB activation: a double-edged sword in health and disease. Cytokine Growth Factor Rev.

[7] Currer R, Van Duyne R, Jaworski E, Guendel I, Sampey G, Das R, Narayanan A, Kashanchi F (2012). HTLV tax: a fascinating multifunctional co-regulator of viral and cellular pathways. Front Microbiol.

[8] Xiao G (2012). NF-kappaB activation: tax sumoylation is out, but what about tax ubiquitination?. Retrovirology.

[9] Pastor MD, Nogal A, Molina-Pinelo S, Meléndez R, Salinas A, González De la Peña M, Martín-Juan J, Corral J, García-Carbonero R, Carnero A, Paz-Ares L (2013). Identification of proteomic signatures associated with lung cancer and COPD. J Proteomics.

[10] Zou Z, Huang B, Wu X, Zhang H, Qi J, Bradner J, Nair S, Chen LF (2013). Brd4 maintains constitutively active NF-κB in cancer cells by binding to acetylated RelA. Oncogene.

[11] Hopewell EL, Zhao W, Fulp WJ, Bronk CC, Lopez AS, Massengill M, Antonia S, Celis E, Haura EB, Enkemann SA, Chen DT, Beg AA (2013). Lung tumor NF-κB signaling promotes T cell-mediated immune surveillance. J Clin Invest.

[12] Chen PM, Wu TC, Wang YC, Cheng YW, Sheu GT, Chen CY, Lee H (2013). Activation of NF-κB by SOD2 promotes the aggressiveness of lung adenocarcinoma by modulating NKX2-1-mediated IKKβ expression. Carcinogenesis.

[13] Schuster M, Annemann M, Plaza-Sirvent C, Schmitz I (2013). Atypical IκB proteins - nuclear modulators of NF-κB signaling. Cell Commun Signal.

[14] Michaux L, Dierlamm J, Wlodarska I, Bours V, Van den Berghe H, Hagemeijer HA (1997). t(14;19)/BCL3 rearrangements in lymphoproliferative disorders: a review of 23 cases. Cancer Genet Cytogenet.

[15] Hsu LJ, Schultz L, Mattison J, Lin YS, Chang NS (2005). Cloning and characterization of a small-size peptide Zfra that regulates the cytotoxic function of tumor necrosis factor by interacting with JNK1. Biochem Biophys Res Commun.

[16] Dudekula S, Lee MH, Hsu LJ, Chen SJ, Chang NS (2010). Zfra is a small wizard in the mitochondrial apoptosis. Aging (Albany NY).

[17] Ando M, Kawazu M, Ueno T, Fukumura K, Yamato A, Soda M, Yamashita Y, Choi YL, Yamasoba T, Mano H (2013). Cancer-associated missense mutations of caspase-8 activate nuclear factor-κB signaling. Cancer Sci.

[18] Suzanne M, Steller H (2013). Shaping organisms with apoptosis. Cell Death Differ.

[19] Maghsoudi N, Zakeri Z, Lockshin RA (2012). Programmed cell death and apoptosis–where it came from and where it is going: from Elie Metchnikoff to the control of caspases. Exp Oncol.

[20] Adrain C, Slee EA, Harte MT, Martin SJ (1999). Regulation of apoptotic protease activating factor-1 oligomerization and apoptosis by the WD-40 repeat region. J Biol Chem.

[21] Edelmann B, Bertsch U, Tchikov V, Winoto-Morbach S, Perrotta C, Jakob M, Adam-Klages S, Kabelitz D, Schutze S (2011). Caspase-8 and caspase-7 sequentially mediate proteolytic activation of acid sphingomyelinase in TNF-R1 receptosomes. EMBO J.

[22] Chandrasekar B, Vemula K, Surabhi RM, Li-Weber M, Owen-Schaub LB, Jensen LE, Mummidi S (2004). Activation of intrinsic and extrinsic proapoptotic signaling pathways in interleukin-18-mediated human cardiac endothelial cell death. J Biol Chem.

[23] Grell M, Zimmermann G, Gottfried E, Chen CM, Grünwald U, Huang DC, Wu Lee YH, Dürkop H, Engelmann H, Scheurich P, Wajant H, Strasser A (1999). Induction of cell death by tumour necrosis factor (TNF) receptor 2, CD40 and CD30: a role for TNF-R1 activation by endogenous membrane-anchored TNF. EMBO J.

[24] Terry Powers JL, Mace KE, Parfrey H, Lee SJ, Zhang G, Riches DW (2010). TNF receptor-1 (TNF-R1) ubiquitous scaffolding and signaling protein interacts with TNF-R1 and TRAF2 via an N-terminal docking interface. Biochemistry.

[25] Ramaswamy M, Cleland SY, Cruz AC, Siegel RM (2009). Many checkpoints on the road to cell death: regulation of Fas-FasL interactions and Fas signaling in peripheral immune responses. Results Probl Cell Differ.

[26] Tabibzadeh S, Zupi E, Babaknia A, Liu R, Marconi D, Romanini C (1995). Site and menstrual cycle-dependent expression of proteins of the tumour necrosis factor (TNF) receptor family, and BCL-2 oncoprotein and phase-specific production of TNF alpha in human endometrium. Hum Reprod.

[27] Strasser A, Jost PJ, Nagata S (2009). The many roles of FAS receptor signaling in the immune system. Immunity.

[28] Hedrych-Ozimina A, Behrendt K, Hao Z, Pofahl R, Ussath D, Knaup R, Krieg T, Haase I (2010). Enhanced contact allergen- and UVB-induced keratinocyte apoptosis in the absence of CD95/Fas/Apo-1. Cell Death Differ.

[29] Matthews GM, Newbold A, Johnstone RW (2012). Intrinsic and extrinsic apoptotic pathway signaling as determinants of histone deacetylase inhibitor antitumor activity. Adv Cancer Res.

[30] Shembade N, Harhaj NS, Parvatiyar K, Copeland NG, Jenkins NA, Matesic LE, Harhaj EW (2008). The E3 ligase Itch negatively regulates inflammatory signaling pathways by controlling the function of the ubiquitin-editing enzyme A20. Nat Immunol.

[31] O’Donnell MA, Ting AT (2010). Chronicles of a death foretold: dual sequential cell death checkpoints in TNF signaling. Cell Cycle.

[32] Tokunaga F, Iwai K (2012). Linear ubiquitination: a novel NF-κB regulatory mechanism for inflammatory and immune responses by the LUBAC ubiquitin ligase complex. Endocr J.

[33] Bernard D, Quatannens B, Vandenbunder B, Abbadie C (2001). Rel/NF-kappaB transcription factors protect against tumor necrosis factor (TNF)-related apoptosis-inducing ligand (TRAIL)-induced apoptosis by up-regulating the TRAIL decoy receptor DcR1. J Biol Chem.

[34] Park YC, Ye H, Hsia C, Segal D, Rich RL, Liou HC, Myszka DG, Wu H (2000). A novel mechanism of TRAF signaling revealed by structural and functional analyses of the TRADD-TRAF2 interaction. Cell.

[35] Ndebele K, Gona P, Jin TG, Benhaga N, Chalah A, Degli-Esposti M, Khosravi-Far R (2008). Tumor necrosis factor (TNF)-related apoptosis-inducing ligand (TRAIL) induced mitochondrial pathway to apoptosis and caspase activation is potentiated by phospholipid scramblase-3. Apoptosis.

[36] Li L, Soetandyo N, Wang Q, Ye Y (2009). The zinc finger protein A20 targets TRAF2 to the lysosomes for degradation. Biochim Biophys Acta.

[37] Arkan MC, Greten FR (2011). IKK- and NF-kappaB-mediated functions in carcinogenesis. Curr Top Microbiol Immunol.

[38] Wei W, Wang D, Shi J, Xiang Y, Zhang Y, Liu S, Liu Y, Zheng D (2010). Tumor necrosis factor (TNF)-related apoptosis-inducing ligand (TRAIL) induces chemotactic migration of monocytes via a death receptor 4-mediated RhoGTPase pathway. Mol Immunol.

[39] Xiao G, Fu J (2011). NF-kappaB and cancer: a paradigm of Yin-Yang. Am J Cancer Res.

[40] Yu C, Argyropoulos G, Zhang Y, Kastin AJ, Hsuchou H, Pan W (2008). Neuroinflammation activates Mdr1b efflux transport through NFkappaB: promoter analysis in BBB endothelia. Cell Physiol Biochem.

[41] Lin Y, Ryan J, Lewis J, Wani MA, Lingrel JB, Liu ZG (2003). TRAF2 exerts its antiapoptotic effect by regulating the expression of Krüppel-like factor LKLF. Mol Cell Biol.

[42] Yang HJ, Youn H, Seong KM, Jin YW, Kim J, Youn B (2013). Phosphorylation of ribosomal protein S3 and antiapoptotic TRAF2 protein mediates radioresistance in non-small cell lung cancer cells. J Biol Chem.

[43] He W, Wang Q, Xu J, Xu X, Padilla MT, Ren G, Gou X, Lin Y (2012). Attenuation of TNFSF10/TRAIL-induced apoptosis by an autophagic survival pathway involving TRAF2- and RIPK1/RIP1-mediated MAPK8/JNK activation. Autophagy.

[44] Bonizzi G, Karin M (2004). The two NF-kappaB activation pathways and their role in innate and adaptive immunity. Trends Immunol.

[45] Bradley JR, Pober JS (2001). Tumor necrosis factor receptor-associated factors (TRAFs). Oncogene.

[46] Wajant H, Henkler F, Scheurich P (2001). The TNF-receptor-associated factor family: scaffold molecules for cytokine receptors, kinases and their regulators. Cell Signal.

[47] Hayden MS, Ghosh S (2008). Shared principles in NF-kappaB signaling. Cell.

[48] Baud V, Karin M (2001). Signal transduction by tumor necrosis factor and its relatives. Trends Cell Biol.

[49] Yeh WC, Shahinian A, Speiser D, Kraunus J, Billia F, Wakeham A, de la Pompa JL, Ferrick D, Hum B, Iscove N, Ohashi P, Rothe M, Goeddel DV, Mak TW (1997). Early lethality, functional NF-kappaB activation, and increased sensitivity to TNF-induced cell death in TRAF2-deficient mice. Immunity.

[50] Tada K, Okazaki T, Sakon S, Kobarai T, Kurosawa K, Yamaoka S, Hashimoto H, Mak TW, Yagita H, Okumura K, Yeh WC, Nakano H (2001). Critical roles of TRAF2 and TRAF5 in tumor necrosis factor-induced NF-kappa B activation and protection from cell death. J Biol Chem.

[51] Grech AP, Amesbury M, Chan T, Gardam S, Basten A, Brink R (2004). TRAF2 differentially regulates the canonical and noncanonical pathways of NF-kappaB activation in mature B cells. Immunity.

[52] Zhang L, Blackwell K, Thomas GS, Sun S, Yeh WC, Habelhah H (2009). TRAF2 suppresses basal IKK activity in resting cells and TNFalpha can activate IKK in TRAF2 and TRAF5 double knockout cells. J Mol Biol.

[53] Zhang L, Blackwell K, Shi Z, Habelhah H (2010). The RING domain of TRAF2 plays an essential role in the inhibition of TNFalpha-induced cell death but not in the activation of NF-kappaB. J Mol Biol.

[54] Chiang MF, Chou PY, Wang WJ, Sze CI, Chang NS (2013). Tumor suppressor WWOX and p53 alterations and drug resistance in glioblastomas. Front Oncol.

[55] Hong Q, Hsu LJ, Chou PY, Chou YT, Lu CY, Chen YA, Chang NS (2013). Self-aggregating TIAF1 in lung cancer progression. Translation Res Med.

[56] Teng CC, Yang YT, Chen YC, Kuo YM, Sze CI (2013). Role of WWOX/WOX1 in Alzheimer’s disease pathology and in cell death signaling. Front Biosci (Schol Ed).

[57] Ghebrehiwet B, Hosszu KK, Valentino A, Peerschke EI (2012). The C1q family of proteins: insights into the emerging non-traditional functions. Front Immunol.

[58] Fu J, Qu Z, Yan P, Ishikawa C, Aqeilan RI, Rabson AB, Xiao G (2011). The tumor suppressor gene WWOX links the canonical and noncanonical NF-κB pathways in HTLV-I Tax-mediated tumorigenesis. Blood.

[59] Del Mare S, Kurek KC, Stein GS, Lian JB, Aqeilan RI (2011). Role of the WWOX tumor suppressor gene in bone homeostasis and the pathogenesis of osteosarcoma. Am J Cancer Res.

[60] Chang JY, He RY, Lin HP, Hsu LJ, Lai FJ, Hong Q, Chen SJ, Chang NS (2010). Signaling from membrane receptors to tumor suppressor WW domain-containing oxidoreductase. Exp Biol Med (Maywood).

[61] Salah Z, Aqeilan R, Huebner K (2010). WWOX gene and gene product: tumor suppression through specific protein interactions. Future Oncol.

[62] Chang NS, Hsu LJ, Lin YS, Lai FJ, Sheu HM (2007). WW domain-containing oxidoreductase: a candidate tumor suppressor. Trends Mol Med.

[63] Chang NS, Doherty J, Ensign A, Lewis J, Heath J, Schultz L, Chen S-T, Oppermamnn U (2003). Molecular mechanisms underlying WOX1 activation during apoptotic and stress responses. Biochem Pharmacol.

[64] Li MY, Lai FJ, Hsu LJ, Lo CP, Cheng CL, Lin SR, Lee MH, Chang JY, Subhan D, Tsai MS, Sze CI, Pugazhenthi S, Chang NS, Chen ST (2009). Dramatic co-activation of WWOX/WOX1 with CREB and NF-kappaB in delayed loss of small dorsal root ganglion neurons upon sciatic nerve transection in rats. PLoS One.

[65] Bouteille N, Driouch K, Hage PE, Sin S, Formstecher E, Camonis J, Lidereau R, Lallemand F (2009). Inhibition of the Wnt/beta-catenin pathway by the WWOX tumor suppressor protein. Oncogene.

[66] Matteucci E, Bendinelli P, Desiderio MA (2009). Nuclear localization of active HGF receptor Met in aggressive MDA-MB231 breast carcinoma cells. Carcinogenesis.

[67] Chang NS, Doherty J, Ensign A, Schultz L, Hsu LJ, Hong Q (2005). WOX1 is essential for tumor necrosis factor-, UV light-, staurosporine-, and p53-mediated cell death, and its tyrosine 33-phosphorylated form binds and stabilizes serine 46-phosphorylated p53. J Biol Chem.

[68] Chang NS, Doherty J, Ensign A (2003). JNK1 physically interacts with WW domain-containing oxidoreductase (WOX1) and inhibits WOX1-mediated apoptosis. J Biol Chem.

[69] Chang NS, Pratt N, Heath J, Schultz L, Sleve D, Carey GB, Zevotek N (2001). Hyaluronidase induction of a WW domain-containing oxidoreductase that enhances tumor necrosis factor cytotoxicity. J Biol Chem.

[70] Lai FJ, Cheng CL, Chen ST, Wu CH, Hsu LJ, Lee JY, Chao SC, Sheen MC, Shen CL, Chang NS, Sheu HM (2005). WOX1 is essential for UVB irradiation-induced apoptosis and down-regulated via translational blockade in UVB-induced cutaneous squamous cell carcinoma in vivo. Clin Cancer Res.

[71] Aderca I, Moser CD, Veerasamy M, Bani-Hani AH, Bonilla-Guerrero R, Ahmed K, Shire A, Cazanave SC, Montoya DP, Mettler TA, Burgart LJ, Nagorney DM, Thibodeau SN, Cunningham JM, Lai JP, Roberts LR (2008). The JNK inhibitor SP600129 enhances apoptosis of HCC cells induced by the tumor suppressor WWOX. J Hepatol.

[72] Hsu LJ, Hong Q, Schultz L, Kuo E, Lin SR, Lee MH, Lin YS, Chang NS (2008). Zfra is an inhibitor of Bcl-2 expression and cytochrome c release from the mitochondria. Cell Signal.

[73] Hong Q, Hsu LJ, Schultz L, Pratt N, Mattison J, Chang NS (2007). Zfra affects TNF-mediated cell death by interacting with death domain protein TRADD and negatively regulates the activation of NF-kappaB, JNK1, p53 and WOX1 during stress response. BMC Mol Biol.

[74] Chang NS, Schultz L, Hsu LJ, Lewis J, Su M, Sze CI (2005). 17beta-Estradiol upregulates and activates WOX1/WWOXv1 and WOX2/WWOXv2 *in vitro*: potential role in cancerous progression of breast and prostate to a premetastatic state *in vivo*. Oncogene.

[75] Su WP, Chen SH, Chen SJ, Chou PY, Huang CC, Chang NS, Dubey R (2012). WW Domain-containing oxidoreductase is a potential receptor for sex steroid hormones. “Sex Hormones”.

[76] Hong Q, Sze CI, Lin SR, Lee MH, He RY, Schultz L, Chang JY, Chen SJ, Boackle RJ, Hsu LJ, Chang NS (2009). Complement C1q activates tumor suppressor WWOX to induce apoptosis in prostate cancer cells. PLoS One.

[77] Sze CI, Su M, Pugazhenthi S, Jambal P, Hsu LJ, Heath J, Schultz L, Chang NS (2004). Down-regulation of WW domain-containing oxidoreductase induces Tau phosphorylation in vitro. A potential role in Alzheimer’s disease. J Biol Chem.

[78] Wang HY, Juo LI, Lin YT, Hsiao M, Lin JT, Tsai CH, Tzeng YH, Chuang YC, Chang NS, Yang CN, Lu PJ (2012). WW domain-containing oxidoreductase promotes neuronal differentiation via negative regulation of glycogen synthase kinase 3β. Cell Death Differ.

[79] Hsu LJ, Schultz L, Hong Q, Van Moer K, Heath J, Li MY, Lai FJ, Lin SR, Lee MH, Lo CP, Lin YS, Chen ST, Chang NS (2009). Transforming growth factor beta1 signaling via interaction with cell surface Hyal-2 and recruitment of WWOX/WOX1. J Biol Chem.

[80] Lan YY, Wu SY, Lai HC, Chang NS, Chang FH, Tsai MH, Su IJ, Chang Y (2013). WW domain-containing oxidoreductase is involved in upregulation of matrix metalloproteinase 9 by Epstein-Barr virus latent membrane protein 2A. Biochem Biophys Res Commun.

[81] Sudol M, Recinos CC, Abraczinskas J, Humbert J, Farooq A (2005). WW or WoW: the WW domains in a union of bliss. IUBMB Life.

[82] McDonald CB, Buffa L, Bar-Mag T, Salah Z, Bhat V, Mikles DC, Deegan BJ, Seldeen KL, Malhotra A, Sudol M, Aqeilan RI, Nawaz Z, Farooq A (2012). Biophysical basis of the binding of WWOX tumor suppressor to WBP1 and WBP2 adaptors. J Mol Biol.

[83] Lin HP, Chang JY, Lin SR, Lee MH, Huang SS, Hsu LJ, Chang NS (2011). Identification of an in vivo MEK/WOX1 complex as a master switch for apoptosis in T cell leukemia. Genes Cancer.

[84] Nakayama S, Semba S, Maeda N, Aqeilan RI, Huebner K, Yokozaki H (2008). Role of the WWOX gene, encompassing fragile region FRA16D, in suppression of pancreatic carcinoma cells. Cancer Sci.

[85] Suzuki H, Katayama K, Takenaka M, Amakasu K, Saito K, Suzuki K (2009). A spontaneous mutation of the Wwox gene and audiogenic seizures in rats with lethal dwarfism and epilepsy. Genes Brain Behav.

[86] Aqeilan RI, Trapasso F, Hussain S, Costinean S, Marshall D, Pekarsky Y, Hagan JP, Zanesi N, Kaou M, Stein GS, Lian JB, Croce CM (2007). Targeted deletion of Wwox reveals a tumor suppressor function. Proc Natl Acad Sci U S A.

[87] Aqeilan RI, Hassan MQ, de Bruin A, Hagan JP, Volinia S, Palumbo T, Hussain S, Lee SH, Gaur T, Stein GS, Lian JB, Croce CM (2008). The WWOX tumor suppressor is essential for postnatal survival and normal bone metabolism. J Biol Chem.

[88] Chang YC, Chiu YF, Liu PH, Shih KC, Lin MW, Sheu WH, Quertermous T, Curb JD, Hsiung CA, Lee WJ, Lee PC, Chen YT, Chuang LM (2012). Replication of genome-wide association signals of type 2 diabetes in Han Chinese in a prospective cohort. Clin Endocrinol (Oxf).

[89] Kataoka K, Shioda S, Ando K, Sakagami K, Handa H, Yasuda K (2004). Differentially expressed Maf family transcription factors, c-Maf and MafA, activate glucagon and insulin gene expression in pancreatic islet alpha- and beta-cells. J Mol Endocrinol.

[90] Yang L, Liu B, Huang B, Deng J, Li H, Yu B, Qiu F, Cheng M, Wang H, Yang R, Yang X, Zhou Y, Lu J (2013). A functional copy number variation in the WWOX gene is associated with lung cancer risk in Chinese. Hum Mol Genet.

[91] Huang D, Qiu F, Yang L, Li Y, Cheng M, Wang H, Ma G, Wang Y, Hu M, Ji W, Zhou Y, Lu J (2012). The polymorphisms and haplotypes of WWOX gene are associated with the risk of lung cancer in southern and eastern Chinese populations. Mol Carcinog.

[92] Becker S, Markova B, Wiewrodt R, Hoffarth S, Hähnel PS, Pleiner S, Schmidt LH, Breitenbuecher F, Schuler M (2011). Functional and clinical characterization of the putative tumor suppressor WWOX in non-small cell lung cancer. J Thorac Oncol.

[93] Zhang P, Ying L, Xu R, Ge S, Mei W, Li F, Dai B, Lu J, Qian G (2010). Tumor-specific, hypoxia-regulated, WW domain-containing oxidoreductase-expressing adenovirus inhibits human non-small cell lung cancer growth in vivo. Hum Gene Ther.

[94] Kimura M, Takenobu H, Akita N, Nakazawa A, Ochiai H, Shimozato O, Fujimura Y, Koseki H, Yoshino I, Kimura H, Nakagawara A, Kamijo T (2011). Bmi1 regulates cell fate via tumor suppressor WWOX repression in small-cell lung cancer cells. Cancer Sci.

[95] Liu CJ, Shen WG, Peng SY, Cheng HW, Kao SY, Lin SC, Chang KW (2013). miR-134 induces oncogenicity and metastasis in head and neck carcinoma through targeting WWOX gene. Int J Cancer.

[96] Baykara O, Demirkaya A, Kaynak K, Tanju S, Toker A, Buyru N (2010). WWOX gene may contribute to progression of non-small-cell lung cancer (NSCLC). Tumour Biol.

[97] Tsai CW, Lai FJ, Sheu HM, Lin YS, Jan MS, Chen SM, Hsu PC, Huang TT, Huang TC, Sheen MC, Chen ST, Chang WC, Chang NS, Hsu LJ (2013). WWOX suppresses autophagy for inducing apoptosis in methotrexate-treated human squamous cell carcinoma. Cell Death Dis.

[98] Batra S, Balamayooran G, Sahoo MK (2011). Nuclear factor-κB: a key regulator in health and disease of lungs. Arch Immunol Ther Exp (Warsz).

[99] Chen W, Li Z, Bai L, Lin Y (2011). NF-kappaB in lung cancer, a carcinogenesis mediator and a prevention and therapy target. Front Biosci (Landmark Ed).

[100] Shen RR, Hahn WC (2011). Emerging roles for the non-canonical IKKs in cancer. Oncogene.

[101] Deng J, Fujimoto J, Ye XF, Men TY, Van Pelt CS, Chen YL, Lin XF, Kadara H, Tao Q, Lotan D, Lotan R (2010). Knockout of the tumor suppressor gene Gprc5a in mice leads to NF-kappaB activation in airway epithelium and promotes lung inflammation and tumorigenesis. Cancer Prev Res (Phila).

[102] Fraser DA, Arora M, Bohlson SS, Lozano E, Tenner AJ (2007). Generation of inhibitory NFkappaB complexes and phosphorylated cAMP response element-binding protein correlates with the anti-inflammatory activity of complement protein C1q in human monocytes. J Biol Chem.

[103] Swanson-Mungerson M, Bultema R, Longnecker R (2010). Epstein-Barr virus LMP2A imposes sensitivity to apoptosis. J Gen Virol.

[104] Kundu JK, Shin YK, Surh YJ (2006). Resveratrol modulates phorbol ester-induced pro-inflammatory signal transduction pathways in mouse skin in vivo: NF-kappaB and AP-1 as prime targets. Biochem Pharmacol.

