# Supplementary figures and images for: Role of WWOX and NF-κB in lung cancer progression
Source: Transl Respir Med. 2013 Nov 14;1:15. doi: 10.1186/2213-0802-1-15 (PMC4715152; doi:10.1186/2213-0802-1-15)

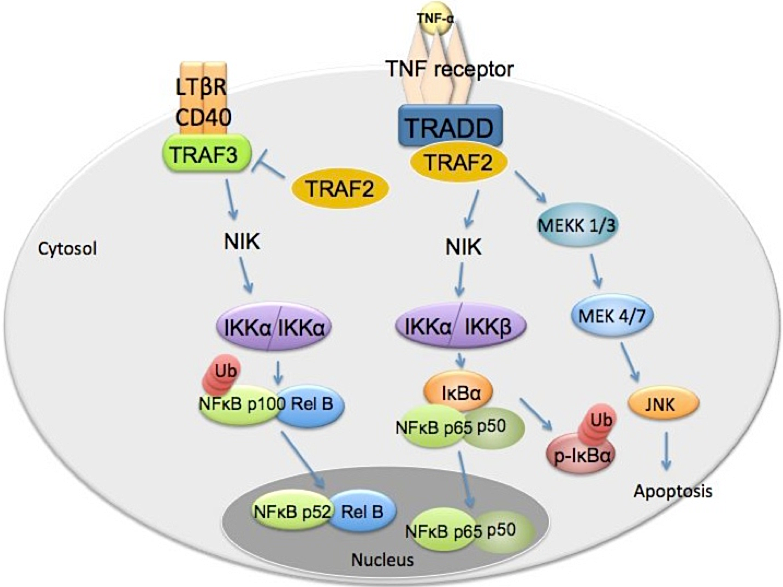

Supplement: Supplementary file 1 — Authors’ original file for figure 1 [file 40247_2013_22_MOESM1_ESM.tiff]

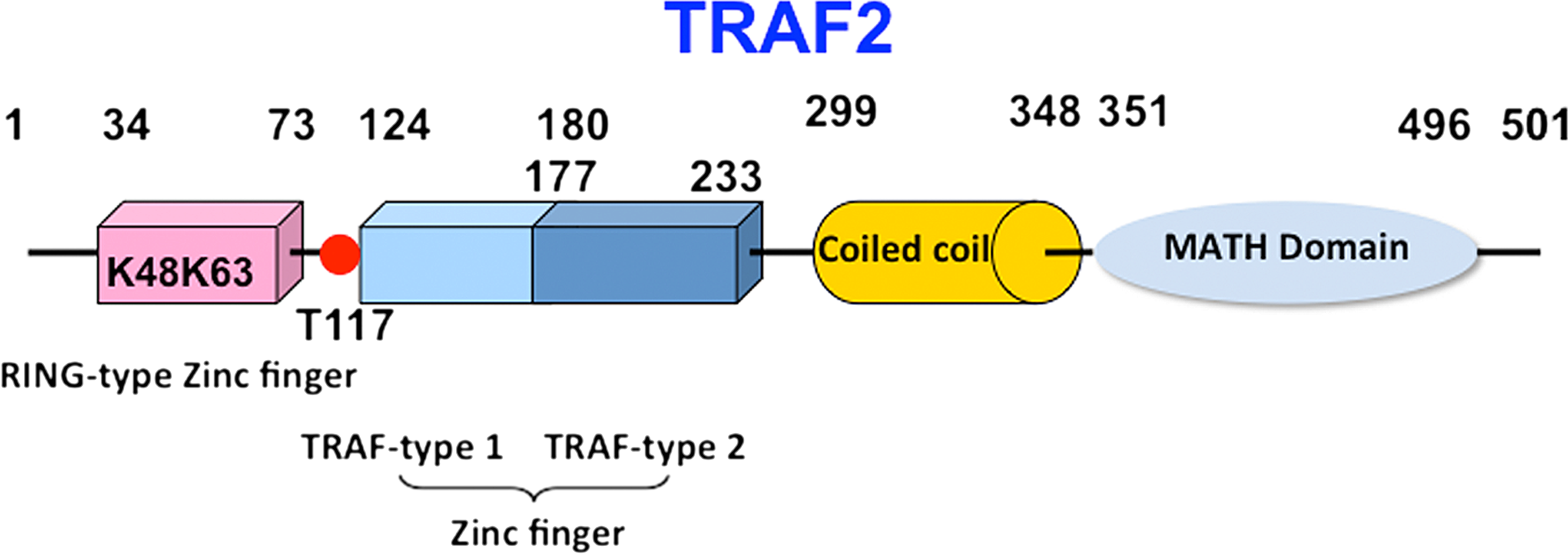

Supplement: Supplementary file 2 — Authors’ original file for figure 2 [file 40247_2013_22_MOESM2_ESM.tiff]

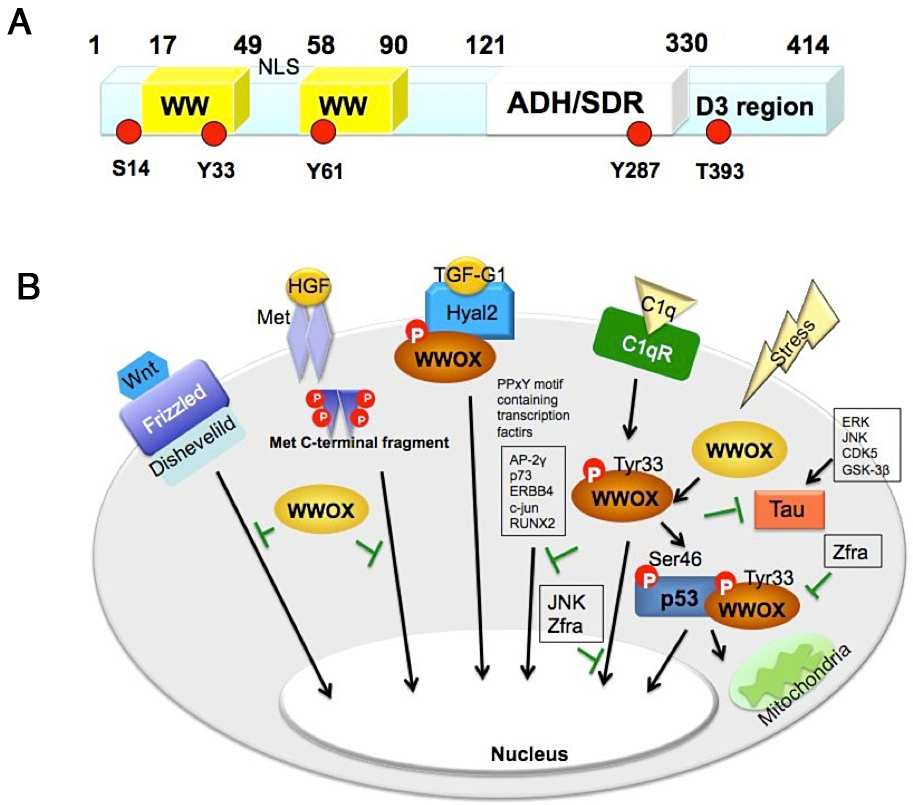

Supplement: Supplementary file 3 — Authors’ original file for figure 3 [file 40247_2013_22_MOESM3_ESM.tiff]
